# Applying User-Centered Design to Develop a Culturally Sensitive, Low-Calorie Meal Plan for Enhancing Dietary Behavioral Control in MASLD

**DOI:** 10.21203/rs.3.rs-7743459/v1

**Published:** 2025-11-19

**Authors:** Maya Balakrishnan, Paola Martinez, Brett Deng, Ivonne Arguelles, Crystal Arguelles, Terri L. Fletcher, Natalia I Heredia, Myriam Ibarra, Anna Christine Rome

**Affiliations:** Baylor College of Medicine; Baylor College of Medicine; Baylor College of Medicine; Baylor College of Medicine; Baylor College of Medicine; Baylor College of Medicine; UTHealth Houston School of Public Health; Baylor College of Medicine; Harris Health System

**Keywords:** fatty liver, health status disparities, diet therapy, dietary intervention, cultural adaptation, ecological momentary assessment

## Abstract

**Background:**

Dietary changes are essential for managing metabolic dysfunction-associated steatotic liver disease (MASLD), yet patients often face barriers related to knowledge, skills, cost, time, and cultural fit. Aims: The purpose of this paper is to report a systematic, replicable method for culturally tailoring a dietary intervention and its application to create a calorie-restricted meal plan for Mexican and Central American patients with MASLD. Guided by the Theory of Planned Behavior and user-centered design, we produced a culturally tailored seven-day structured meal plan aligned with dietary guidelines for MASLD and weight loss.

**Methods:**

We conducted a three-phase, mixed-methods process among Mexican/Central American patients with MASLD from a safety-net healthcare system in Houston, Texas (n = 25). Phase 1 characterized meal patterns and preferences through semi-structured interviews. Phase 2 integrated findings with clinical nutrition guidelines to develop structured meal plan prototypes at 1200-, 1500-, and 1800-calorie levels. Phase 3 tested usability through ecological momentary assessments and daily interviews.

**Results:**

Participants typically consumed home-cooked meals centered on animal protein, legumes and simple grains, with lunch and dinner preparation being most challenging. Recipe modifications focused on increasing fiber and reducing fat and refined carbohydrates. Usability testing showed that participants found the plans culturally aligned and practical, improving portion awareness and dietary self-efficacy.

**Conclusions:**

This study demonstrates a replicable, patient-centered approach to culturally tailoring dietary interventions and provides a concrete product – a calorie- restricted meal plan. Next steps (underway) are to evaluate the meal plan’s larger-scale implementation and impact on dietary change and weight loss.

## INTRODUCTION

Metabolic-dysfunction-associated steatotic liver disease (MASLD) is the most common chronic liver disease, currently affecting over 30% of adults in the United States [1, 2]. Caused by obesity and insulin resistance, it is closely related to metabolic syndrome and associated with cardiovascular complications and progressive liver injury, which can culminate in cirrhosis and liver failure [3)].

Dietary change is central to MASLD treatment. Sustained weight loss of 7–10% is associated with improved liver disease and cardiovascular benefits [4]. To promote weight loss, clinical guidelines emphasize reducing total caloric intake. Guidelines also promote holistic dietary patterns, aligned with Mediterranean-patterned diets, given their independent associations with improved hepatic steatosis and metabolic markers [4]. Lastly, guidelines recommend reducing sugar-sweetened beverages, saturated fat, red and processed meats, and ultra-processed foods, given their associations with increased MASLD risk [6–9].

However, dietary change is difficult. It requires selecting and calculating the right combination of foods and nutrients, planning meals, modifying recipes and cooking methods and eating correct portion sizes. This requires a high degree of perceived behavioral control or self-efficacy, meaning how capable a person feels of achieving healthy eating behaviors. According to the Theory of Planned Behaviors, behavioral control is a strong determinant of a person’s motivation to make behavioral changes, influencing adherence to dietary recommendations [10].

Patients with MASLD and related metabolic conditions frequently report barriers to behavioral control, including inadequate knowledge and skills for healthy eating. Additionally, many perceive the cost and accessibility of healthy foods, along with cultural and family eating practices, as barriers to dietary control [11–17]. Interventions have aimed to address these challenges. Providing preprepared, portion-controlled meals [18, 19] and liquid meal replacements [20]), by reducing reliance on behavioral control, improve weight-loss rates among clinical trial participants. Cooking classes and culinary medicine have enhanced dietary knowledge, self-efficacy and practical skills [21]. However, such interventions are resource-intensive and require continued external funding to sustain. There is a pressing need to develop scalable, cost-effective strategies implementable within routine clinical practice to address behavioral control-related barriers.

A potentially sustainable approach to improving dietary behavioral control is using structured meal plans—dietary tools specifying what and how much to eat daily. These help translate complex nutritional recommendations into practical daily eating behaviors. Compared to standard dietary counseling alone, adding a structured five-day meal plan resulted in 50% and 100% greater weight loss at 6 months and 1 year, respectively, among 163 adult clinical trial participants [22] and improved weight loss among 109 adolescents with obesity[23]. Additional benefits include improved dietary knowledge, more accurate portion sizes, regular meal patterns and reduced snacking.

To address behavioral control among Mexican and Central American (M/CA) patients with MASLD, we developed a culturally tailored, seven-day structured meal plan. We prioritized this population because it has a disproportionately high MASLD burden in the United States [24] and significant representation in our clinical setting in Houston, Texas. The meal plan was conceptualized as a way of enhancing behavioral control by modeling *how* to implement medical nutrition recommendations using familiar, culturally relevant foods [25].

We employed a user-centered design approach guided by nutrition science and the food literacy framework by Vidgen and Gellegos [26]. We hypothesized that this process would yield a feasible, acceptable, culturally congruent meal plan that could enhance behavioral control and thereby improve participants’ ability to implement dietary recommendations. This article details the meal-plan development process, which may be a scalable model for other nutrition-related conditions and patient groups.

## METHODS

**Figure 1:**
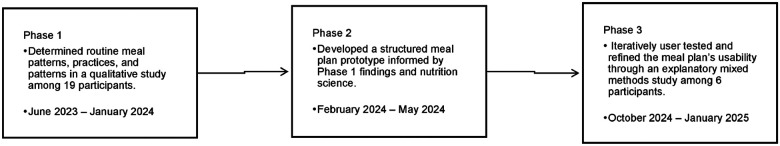
Three-phased user-center design process

We developed the meal plan using a three-phase, patient-centered design process (Fig. 1), adapted from user-centered design, which emphasizes iterative engagement with users to optimize product usability [27]. Our intended users were M/CA patients needing weight loss to manage MASLD, type 2 diabetes or metabolic syndrome. The study was IRB-approved by the relevant university and hospital systems and followed Helsinki Principles.

### Phase 1

Phase 1 was a qualitative study to determine participants’ usual meal preparation, eating habits and composition patterns, using one-time semi-structured interviews.

#### Study Sample

We purposively sampled adults (18–70 years) with M/CA heritage, BMI ≥ 25 kg/m^2^, and a diagnosis of MASLD, type 2 diabetes, and/or metabolic syndrome. Exclusions were advanced cirrhosis, hemoglobin A1C ≥ 9%, serious competing comorbidities or contraindications to calorie reduction. Recruitment occurred through dietitian diabetes and hepatology clinics within a safety-net health system in Houston, Texas. Clinicians introduced the study, and coordinators followed-up with those interested. We obtained verbal consent and offered $20 for interviews. We anticipated needing 15–20 participants to reach data saturation, the point at which data obtained begins to be repetitive, given our study’s focused objectives, standardized interview questions and homogenous study population [28, 29].

#### Data Collection

We developed a semistructured interview guide with input from dietitians experienced in counseling the priority population. It included questions, probes and prompts about cooking practices, meal composition, eating patterns and food preferences, as well as detailed accounts of meals consumed over four days (two weekdays, two weekend days), home-cooked recipes, and common household staples (Appendix A). The guide was pilot-tested and refined with feedback from internal staff and two volunteer prestudy participants.

Two bilingual female research assistants (CA, an undergraduate social science student, and MI, a Master’s-trained public health professional) conducted phone interviews in participants’ preferred language. Both were familiar with M/CA cuisines, qualitative interviewing, and had no prior relationship with participants.

Demographic and clinical data were collected via preinterview survey and electronic medical record review. MASLD, type 2 diabetes, hypertension and dyslipidemia were classified by documented diagnosis or treatment. Acculturation was measured using the Brief Acculturation Scale for Hispanics (score > 3 signifying a higher level of acculturation) [30] and food insecurity via the two-item Hunger Vital Sign (affirmative response to either indicating insecurity) [31].

### Data Analysis

We conducted thematic analysis to identify patterns across participants’ meal preparation, eating practices and preferences that would influence meal plan construction[32]. Interviews were recorded, transcribed and translated from English to Spanish as needed and imported into Atlas.ti 23 (Atlas ti Scientific Software Development GmbH). The research team (MI, CA, BD, MB) reviewed the initial five transcripts to apply deductive codes based on interview questions and develop inductive codes from emerging content [33]. As no new codes emerged in subsequent interviews, we finalized the codebook and applied it to all transcripts. During weekly meetings, the team discussed transcripts and compared and resolved differences in coding. MB initially categorized and organized final codes in a thematic map, which the team iteratively refined. Final themes were structured around key domains important for creating a feasible meal plan. Rigor was maintained through review and discussion of transcripts, codes and themes.

### Phase 2

Phase 2’s objective was to create a prototype of the seven-day meal plan, guided by three main principles. The first was to ground the plan in patterns, practices and preferences emerging through phase 1 interviews. To achieve this, we incorporated recipes for meals emerging frequently across interviews and modified them using foods, ingredients and cooking equipment participants commonly had. Secondly, we constructed calorie-restricted meal plans to support average weight loss of one pound weekly. To this end, we planned one prototype rationed at three standard calorie levels: 1200, 1500, and 1800 calories per day for people with a baseline weight of 54 to 77 kilograms, 78 to 99 kilograms, and > 100 kilograms, respectively. These calorie thresholds have been used across several lifestyle weight-loss interventions, including the Diabetes Prevention and Look AHEAD Lifestyle Programs [34–36]. The third guiding principle was to align meals’ macronutrient distribution and composition with U.S. Department of Agriculture and World Health Organization dietary guidelines [37, 38], relevant for all metabolic syndrome conditions. To this end, we aimed for macronutrient distribution of 50% carbohydrates, 20% protein and 30% fat, with at least 25 grams of dietary fiber per day. In addition, recipe modifications focused on incorporating complex carbohydrates (e.g., whole grains, lentils and legumes), lean protein sources (e.g., fish, chicken, beef), fresh vegetables and fruits, and plant-based oils. ACR, a licensed dietitian, modified recipes using Nutritionist Pro^™^ Diet Analysis software (Axxya Systems LLC, Redmond, WA).

### Phase 3

In phase 3, we assessed the meal plan’s usability among participants with surveys and interviews in a mixed-methods explanatory study design. The primary objective was to identify and fix major usability problems.

#### Study Sample

We purposively recruited M/CA patients with BMI ≥ 25kg/m2 and MASLD who were not in Phase 1 and were willing to use the seven-day meal plan, applying Phase 1’s exclusion criteria and recruitment approach. The objective was iterative user testing, refining the prototype after each participant and concluding data collection when two consecutive participants reported no major usability problems. Based on published user testing guidance—indicating five users can detect 85% of major usability problems during conceptual user testing—we anticipated needing five participants to achieve our objective [39]. We obtained written informed consent and offered $120 to participate in the seven-day study.

#### Data Collection

Participants were assigned meal-plan calorie levels based on their weight and instructed to follow the plan as written for seven days, except for: (1) modifying cooking to improve palatability (e.g., adjusting seasonings, cooking times) and (2) substituting ingredients due to unavailability, allergy or strong dislike. In both cases, they were asked to maintain original portion sizes, using plan guidance.

**Figure 2:**
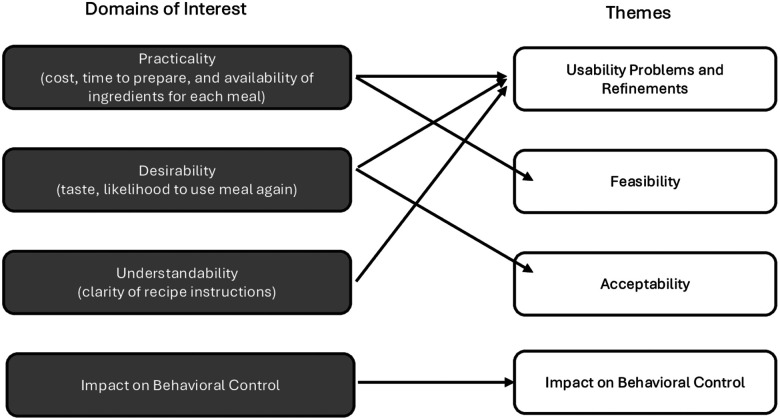
How phase 3’s domains of interest informed themes

We evaluated four domains (Fig. 2): *practicality, desirability, understandability* and *impact on dietary behavioral control*. Usability problems were assessed based on the first three domains, based on the University of South Carolina’s Drivers of Food Choice framework [40], Vigden’s food literacy model [26], and research on factors influencing meal plan usage [41]. Feasibility and acceptability were assessed using the practicality and desirability domains, respectively.

We used ecological momentary assessment (EMA) surveys and daily semistructured interviews (Appendix B). Each participant was scheduled to complete 21 EMA surveys (3 per day) and 7 interviews. The 11-item EMA survey collected structured feedback about each meal’s cost, preparation time, taste and acceptability in real time [42] and was sent via REDCAP to reach participants three times around their typical reported mealtimes [43]. A single text reminder was sent if surveys were not completed within one hour. Study team members and three patient volunteers (not study participants) reviewed and refined survey items for face validity.

Daily interviews assessed user experiences, followed-up all EMA responses, deeply probed EMA responses indicating low usability or negative reactions and solicited potential improvements. The interview guide covered the three usability domains for meals prepared on the prior day and, on the last day, additionally explored the plan’s impact on eating perceptions and behaviors (Appendix B). Two bilingual female research assistants (IA and PM, both with Bachelor’s degrees in biological sciences) familiar with M/CA cuisines and qualitative interviewing, conducted phone interviews in participants’ preferred language.

### Data Analysis

We used descriptive statistics (counts, proportions) to summarize EMA responses. Rapid qualitative analysis of interview data was used to generate targeted, actionable insights for refining the meal plan, assessing feasibility and acceptability and exploring its impact on behavioral control [44].

Interviews were recorded, transcribed, and translated as needed. Using thematic analysis and the matrix method, we organized data using four prespecified domains (practicality, desirability, understandability and behavioral control), with a fifth domain added to document suggestions to fix emerging major meal-plan problems [44].

For the first two participants, the team (PM, IA, MB) independently reviewed and summarized all 14 interviews, discussing them daily and compiling them into a shared matrix, which they then finalized. Subsequent interviews were summarized using the finalized matrix by one team member and reviewed by the interviewer. Summaries and quotations were organized using Microsoft Excel, with a matrix with rows for participants and columns for domains. The team met several times a week to reconcile discrepancies, flag usability concerns and agree on plan refinements. Data were ultimately grouped into four main themes: meal plan usability problems, feasibility, acceptability and behavioral impact, as depicted in Fig. 2. Meal Plan Refinements

After participants completed the seven-day usability testing, the analysis team and dietitian (ACR) reviewed emergent usability problems to determine whether major, minor, or no changes were needed. *Major problems* necessitated changes to recipe ingredients, preparation steps or written instructions. *Minor problems* could be addressed by how future users would be counseled about the meal plan — either through introductory instructions or verbal guidance. No changes were made in response to feedback based solely on personal preferences. Refinements were made by consensus, incorporating participant input and dietitian expertise; and the updated meal plan was tested by the next user.

## RESULTS

### Phase 1 – Meal Preparation Patterns and Preferences

Phase 1 included 19 participants (see Table 1). All were foreign born; most had low acculturation levels. Eleven women and two men were primarily or partially responsible for home meals; the rest relied entirely on a female household member to prepare home meals. Median cost of household groceries was $200 weekly. Some risked food insecurity. Interviews lasted a median of 63 minutes (range 42–86). No new data emerged after interview 16, confirming saturation.

Three main themes with implications for structuring a meal-plan prototype emerged: source of meals (where meals were prepared or obtained), meal frequency and meal composition/preferences. We present each with supporting quotes. Additionally, we inventoried common homemade meal recipes that could be incorporated into the prototype and foods and staples (animal proteins, vegetables, fruits, legumes, grains, cooking oils and appliances) commonly used by participants appropriate for recipe modifications in phase 2 (Supplementary Table 2).

#### Source of Meals

Most participants predominantly ate home-cooked meals prepared for the entire household, describing this as part of traditional family life. A few had shifted from regularly eating “outside meals” to “home meals” for health and cost reasons: “When [my health] was okay, and I had my job, I used to eat out at restaurants [often] … but now I don’t … there’s no money and I’m ill” (P10/55-yoM with diabetes).

Some occasionally ate out – typically on weekends – for a change, to “take a break” from cooking or for social activities like celebrations or church. As a 42-yo mother of two explained, “Usually, everything is from home, but on the weekend [we eat out because] you get tired spending so much time in the kitchen”. A few habitually ate weekday lunches, typically fast food, from outside because they found it difficult to prepare or carry a meal to work. “Sometimes I am in places where there is no food. I need to get something fast. .. I am a driver, so if it takes one or two hours and then I’m screwed” (P19/48-yoM).

A minority predominantly ate outside meals, often alone or with nonhousehold members, typically store or restaurant bought or occasionally from a friend’s home. They did not cook for a variety of reasons” “I just get lazy” (P4/56-yoF, notary with three adult children) or “because it’s just for me.” (P1/42-yoF, housekeeper with one adult child). Others additionally cited preference and practicality: “I hardly cook at home because I work a lot…Also, [outside food] tastes good, it’s cheap, and it’s cooked (P12/35-yoF, driver with two children).

#### Meal Frequency

Participants habitually ate two or three meals a day. “Three-meal-a-day” eaters typically ate discrete morning, afternoon and evening meals. A few who woke up late in the day or worked late or night shifts, maintained a “three-meal-a-day pattern” at shifted times, for example, in afternoon, evening, and nighttime. On weekends, this pattern was often disrupted with a preference for two larger meals in late morning/early afternoon and evening, timed around social or family activities.

“Two-meal-a-day” eaters typically skipped breakfast or lunch, eating one “heavy” meal, a second smaller meal, and snacks during the day. For some this was simply a longtime preference: “I usually don’t have breakfast. In the morning … the first meal I eat is lunch” (P5/62-yoF, homemaker). Others were too busy for three meals: “I make only two meals because I don’t have time… we make homemade bread to sell: I get up at 5AM, we have breakfast at 9AM, and sometimes we don’t have time to eat until night when we’re done” (P7/homemaker).

#### Meal Composition and Preferences

Because the objective of this phase was to characterize homemade meals for adaptation, we focused on homemade meal composition and preferences. Nearly every meal featured an animal-based protein as its primary ingredient: most frequently, eggs for breakfast and chicken or beef for lunch and dinner. Ground turkey, ham deli meats as part of sandwiches, seafood and fish featured among some participants’ meals, but less frequently.

Legumes – most commonly pinto, red, or black beans – often accompanied meals as sides. Non-starchy vegetables (e.g., broccoli, cabbage, squash) were either absent or included in small portions as a side or incorporated into an animal protein-based dish. The most frequently consumed grains were rice, tortillas and bread. Cheese and cream were frequent dairy-based condiments, added for taste.

Participants favored quick, simple meals: “We cook the most regular or simple meals here at the house. .. caldo, picadillo, pozole, tamales, enchiladas. We do all those things easily and quickly (p7/homemaker, cooks for household of six)”. They favored chicken- or turkey-based lunches and dinners, as they were quicker to prepare than beef- and pork-based. “Meat is difficult because it takes time. It takes like four hours. I do what’s faster, like turkey or chicken sausages (p6/42-yoF homemaker, cooks for household of five).

Lunch and dinner were the most difficult to prepare. For some, lunch was difficult because of challenges preparing and then carrying food to work. Several mothers felt simultaneously juggling meal preparation with childcare was challenging. “Dinner is difficult … my children arrive from school hungry and want a heavy meal that makes them feel full. So it’s always difficult for me to think what I can prepare for dinner and is also healthy for me (P16/48-yoF, three children).

Generally, participants described liking fruit, vegetables, fish and seafood. Cost was mainly a barrier to purchasing seafood and fruit, as one 35-yo F observed, I love grilled shrimp … rice, asparagus, salmon. I love baked salmon fillet with asparagus and zucchini. I prepare it, but the main thing that stops me is the price. For a fillet of salmon, it’s $25; and for a package of wieners, you pay $1.50, so you grab the wieners, instead. (P12/35-yoF)

### Phase 2 – Meal Plan Prototype

#### Meal Plan Structure and Options

Because Phase 1 participants described a two- (one light and one heavy) or three-meal- a-day pattern, we offered three meal options a day (breakfast, lunch and dinner) of roughly equivalent calorie compositions and one low-calorie snack. This allowed “two-meal-per-day” eaters to eat double the portion of one meal and a single portion of a second meal, while maintaining their prescribed calorie count.

First, we created a prototype skeleton populated with the primary animal protein that would characterize each of the 21 meals offered (3 meals a day). Then we identified recipes frequently reported in phase 1 that used or could be modified to use the protein. Phase 1 information regarding meal composition, preferences and typical availability at home shaped our decision to incorporate eggs into several breakfast items and chicken and turkey into several lunch and dinner items. Though included, we limited meals that that incorporated seafood (due to cost concerns in phase 1) and beef (due to time concerns expressed in phase 1). The prototype included two entirely plant-based meals and three meals (two sandwiches and one taco) that could be quickly prepared and carried to work. We created seven snacks (one per day) using protein sources (cheese, nuts, peanut butter) and fresh fruits reported in Phase 1.

#### Recipe Modifications

Recipe modifications were geared toward achieving the three planned calorie levels (1200, 1500, and 1800 calories/day) and nutrient composition. The most frequently required modifications were reducing fat, changing carbohydrate composition, and increasing portions of high-fiber vegetables in the original recipes. Fat content was reduced by lowering the amount of meat, cheese and avocado in the original recipe and by switching cooking methods from frying to sautéing, baking, or boiling. Complex carbohydrate sources of original recipes – such as rice, corn, beans and tortillas – were retained but in smaller portions. Simple carbohydrates, such as foods and drinks with added sugars (e.g., pan de dulce, sweet teas) were eliminated. Portions of high-fiber vegetables commonly reported in phase 1 (e.g., carrots, squash, green beans, peppers and broccoli) were increased with instructions that they fill half the plate or approximately one cup per meal. Except for dairy- and/or fat-based condiments (e.g., creams, cheese, avocado), original recipes’ condiments and flavorings were retained. One example of recipe modification is traditional chicken flautas. In the original recipe, chicken is wrapped in a tortilla and fried; in the modified version, the amount of meat is reduced, the dish is baked instead of fried, one cup of vegetables as a salad is added, and the serving size of flautas portioned to limit calories consumed.

### Phase 3 – Meal Plan User Testing

Phase 3’s user testing population included six women, median age 51y and BMI 35 kg/m2 (Table 1). All were foreign-born with low acculturation levels, and the median household grocery cost was $160 weekly. Three were at risk for food insecurity.

Based on their weight, three were assigned and tested the 1,800-calorie/day meal plan, two the 1500-calorie/day meal plan, and one the 1200-calorie/day plan. Of 21 meals in the plan, participants collectively prepared 119 meals: three prepared all 21, two prepared 20, and one prepared 16. The EMA response rate was 89% (Supplementary Table 2). Each participant completed seven daily interviews (median 20 minutes, range 9–41 min). Findings are presented in four sections: user problems and refinements, feasibility, acceptability and impact on behavioral control (Fig. 2). No major usability problems requiring refinements emerged after participant 3, confirming saturation.

#### Usability Problems and Prototype Changes

Major problems with the meal plan mainly involved understandability and desirability, resolved with input from participants one through three, and ceased thereafter (Supplementary Table 3). Major understandability problems included a confusing meal plan layout, recipe names not making sense, and a few confusing cooking instructions. In response, we reorganized the plan’s layout, changed recipe titles and clarified cooking instructions.

There were two types of major desirability problems. The first was a need to substitute the animal protein, complex carbohydrate, or legume called for by a recipe – because of intolerance (e.g., seafood allergy), taste preference (e.g., replacing turkey or chicken with beef or rice with pasta), or familiarity (e.g., replacing red beans with black or pinto beans). Secondly, in a few instances, recipe instructions did not produce a palatable meal. In response, we developed user instructions on how to make isocaloric protein, complex carbohydrates, legume substitutions and improved recipes, using participant feedback.

Two minor problems emerged regarding desirability. First, participants suggested or made meal modifications that would significantly increase fat and calorie content, such as adding avocado, cream, or substituting oatmeal flour for corn flour. In response, we noted the need to emphasize, during both verbal and written counseling, that such modifications can significantly alter the intended calorie content. Secondly, 1200- and 1500-calorie/day plan users felt some meals were too light or left them hungry. In response, we reinforced counseling in the prototype guiding users to add nonstarchy vegetables to meals as a low-calorie strategy to increase meal volume and satiety.

#### Feasibility

Participants found meals’ costs, preparation times, and ingredient availability feasible. They estimated meals cost $2 to $30, with “agreed” or “strongly agreed” across 98% of EMA responses regarding cost acceptability. Qualitative probing of less positive responses (Supplementary Table 2) revealed that they still found costs acceptable, attributing higher expenses to meat, seafood purchases and preparation for multiple family members.

Meal preparation times ranged 5 to 30 minutes for breakfasts, 10 to 120 minutes for lunches, and 5 to 120 minutes for dinners. Most found these acceptable, noting that cooking animal proteins (especially chicken and beef), preparing legumes and rice, and measuring ingredients were the most time-consuming tasks.

Participants typically had all necessary ingredients. When unavailable, they substituted or omitted them without difficulty. Several substituted available cheeses when specific cheeses were not available. Commonly missing was turkey, which two participants substituted with another animal protein, and one purchased for testing and reported liking. Other less commonly available ingredients included mushrooms, sesame oil, mustard and balsamic vinegar. A few did not have spicy condiments such as jalapeños, serrano peppers, black pepper, and pepper flakes because of personal preferences.

I have everything at home. These recipes you’re providing use what we [Hispanics] all have at home, especially when it comes to the meat. What I don’t usually have is turkey but always have chicken, beef, milanesa, and all the vegetables. (P3/67-yoF)

#### Acceptability

EMA responses across most meals indicated participants agreed or strongly agreed that meals tasted good (98%) and they would reuse the recipe (95%, Supplementary Table 2). Participants universally felt the meals were familiar and acceptable to their families. “Well, this is how I cook. I mean, you’re not giving me recipes that I don’t normally make for myself and my kids. They can also eat this” (P5/49-yoF).

They also liked exposure to new flavors: “The recipes I’m seeing are something I’m learning from, too; and I’m experimenting with flavors“ (P2/42-yoF).

Qualitative probing of five “neutral,” “disagree,” or “strongly disagree” responses to reuse the recipe revealed major problems addressed in the refinements. It also uncovered, mainly among lower-calorie-meal-plan (1200 and 1500 calories/day) users, challenges adjusting to the higher vegetable and lower animal protein portions and hunger after some meals. For example, P6 described, It was difficult to follow this meal plan. I wasn’t used to it. I would try to stick with it because it has helped me; I feel like I lost weight. … but it was difficult just eating mostly vegetables… and I thought the [portions of protein] was very little.

Participants felt adhering to the meal plan would ultimately require “mentally adjusting, knowing that this is your diet” (P5).

#### Impact on Behavioral Control

Two key subthemes emerged regarding the impact of the meal plan on behavioral control (Supplementary Table 6). First, participants felt the meal plan reframed how they thought about normal portion sizes and came to realize they had previously been overeating. P3 observed, I’m now seeing the actual amounts that I should eat, I usually cook for five or six people … And you don’t measure portions, you just eyeball it. And, I take portions that are more than I should eat. .. now I realize what I actually should be eating.

Secondly, participants felt the plan taught them healthier cooking strategies: how to reduce fat, reduce frying, add vegetables and incorporate lean proteins. They all felt that several meals were typical ones that they prepare, but using the meal plan taught them how they could modify other recipes using healthier substitutions and methods. For instance, P4 expressed, “I would like to know what meats I can eat to reduce fat in my liver,” and appreciated that the meal plan provided a way to prepare alternative, leaner animal proteins, like turkey, that she had not previously been exposed to. Likewise, P3 felt “[with this recipe] I can eat less red meat and make a chicken picadillo. .. it’s better to eat chicken and fish.”

## DISCUSSION

Despite widespread recognition of the need for culturally tailored dietary interventions, practical guidance on how to design them remains limited; this paper addresses that gap by detailing a replicable, user-centered process for developing a structured meal plan for M/CA patients with MASLD. Early conceptual testing suggests our process has yielded a feasible, acceptable, and usable meal plan. Qualitative assessment of the meal plan indicates it enhances key aspects of behavioral control, such as recognizing proper portion sizes and learning how to improve dietary quality of meals.

### The Process

The central strength of our design was integrating participant input at every stage, ensuring the intervention was grounded in their experiences and needs. Guided by established food choice frameworks and previous research [26, 40, 41], the process also addressed key domains of food-related behaviors and preferences. In this first iteration of the process, we relied heavily on qualitative methods for several reasons. The first was to gain deep insight into the priority population’s eating patterns and preferences. We additionally were able to distill three key aspects of eating patterns and practices important for constructing a meal plan: meal frequency, meal composition and typical staples at home. Phase 1 findings align with patterns, preferences and composition of meals reported by other qualitative studies among similar populations, supporting validity of the approach [45, 46]. We used EMAs in phase 3 to enhance ecological validity by capturing reactions to the meal plan in participants’ natural environments and to minimize recall bias by capturing in-the-moment responses on taste, convenience and satisfaction [47, 48]. Extensive qualitative probing allowed us to confirm and obtain additional in-depth explanations of user EMA reactions to meals’ desirability and practicality. Phase 3 findings that the meal plan was feasible and acceptable among users suggest that the participatory design process effectively incorporated the priority population’s preferences.

While promising, the design process should be tested in diverse patient populations with different dietary needs to assess broader applicability. It is important also to keep in mind that, to take a group approach to intervention development, the process has to be implemented among a population sharing sociocultural patterns and requires that intervention developers possess a priori knowledge about this. In its current form, the design process took 18 months. To improve efficiency, future iterations could incorporate streamlined data-collection techniques rather than the extensive qualitative methods we used in this first iteration. For example, phase 1 could use an initial survey to understand meal timings, typical staples and frequently prepared homemade recipes, with interviews reserved for clarifying details. Incorporating generative artificial intelligence into Phase 2 recipe modifications could produce a prototype more efficiently. Phase 3 user testing could be made more efficient by refining the EMA surveys to collect all practicality (cost, time to prepare meals) and initial desirability information (taste, likelihood to prepare meal again) with interviews reserved solely for improving product usability in event of negative EMA reactions.

### The Meal Plan

Our qualitative findings of improved behavioral control among meal plan users aligns with prior research. Structured meal plans – through meal planning and specific portion size guidance – are thought to reduce the cognitive load associated with dietary decisions and thereby, facilitate dietary adherence [22, 49]. It should be noted that this structured meal plan is, by design, specifically tailored for, and thus limited to, M/CA patients who eat traditional home-cooked meals. Thus, this intervention would be inappropriate for populations more highly acculturated or not willing to cook. However, the design process presented here could be used to develop an appropriately tailored intervention for other populations. Larger-scale testing –currently ongoing in a pilot feasibility trial among 50 participants [50] – is necessary to determine actual meal plan usage and long-term dietary adherence and may uncover additional usability problems missed in smaller-scale testing. Future effectiveness testing is ultimately required to assess the meal plan’s impact on behavioral control and long-term dietary behaviors among M/CA patients.

## CONCLUSIONS

This study offers a replicable, patient-centered process for developing culturally tailored meal plans to support dietary change in populations with chronic metabolic conditions. It highlights the value of embedding behavioral science and qualitative methods into intervention design. Clinically, the meal plan we developed provides a low-cost, scalable tool to address barriers to behavioral control among M/CA patients.

## Supplementary Material

This is a list of supplementary files associated with this preprint. Click to download.

- SuppT1.HomeStaples.docx
- SuppT2.EMAResponses.docx
- SuppT3.UserFeedbackChanges.docx
- SuppT4.Feasabilityquotes.docx
- SuppT5.AcceptabilityQuotes.docx
- SuppT6.BehaviorImpactQuotes.docx
- Supp.AppendixAB.SurveysInterviewGuidesfinal.docx
- SuppFig1tiff.tiff

## Figures and Tables

**Table 1:** Participant Characteristics

	Phase 1	Phase 3
**sample size**	19	6
**Age (years)**, median (range)	48 (35–62)	51 (42–73)
**Gender (Female) N**	12	6
**BMI (kg/m^2^)**, median (range)	32 (25–62)	37 (26–43)
**Medical Conditions** *		
MASLD	16	6
Type 2 Diabetes	16	5
Metabolic syndrome	15	4
**Birth Country**		
Mexico	10	4
El Salvador	7	1
Guatemala	1	1
Honduras	1	0
**Acculturation status**		
Low (acculturation score ≤ 3)	18	6
High (acculturation score > 3)	1	0
**Years in the US**, median (range)	26 (10–42)	29 (24–30)
**Marital Status**		
Married/domestic partner	12	4
Separated/Divorced	4	2
Single/never married	2	0
Widowed	1	0
**Household Count**, median (range)	4 (2–11)	4 (range 2–7)
**Number of children<18yrs in household**, median (range)	3 (0–5)	2 (0–3)
**Occupation**		
Unemployed	0	2
Homemaker	6	1
House Cleaner	4	3
Yard Worker	2	0
Restaurant Worker	2	0
Driver	3	0
Other (white collar job: notary, football agency)	2	0
**Food insecurity**		
at risk	5	3
not at risk	12	3
**Weekly grocery bill**, median (range)	$200 ($80–$400)	$160 ($125–$225)
**Person responsible for meal preparation at home**		
Participant entirely or partially responsible	11	6
Participant’s spouse	3	0
Other member of participant household**	5	0

Results are reported as number with the characteristic unless otherwise specified.

* Number of participants with the medical condition reported. These are not expected to add up to total number.

** Participants’ adult daughter, sister, mother-in-law

## Data Availability

The full individual-level survey responses, qualitative transcripts, and audio recordings from this study contain sensitive participant information, and therefore cannot be made publicly available in order to protect confidentiality. Aggregated data—including survey responses by question, thematic summaries, and anonymized exemplar quotations—are provided in the Supplementary Materials. De-identified summary tables beyond what is included may be shared by the corresponding author upon reasonable request, subject to approval by the ethics committee and/or institutional review board.

## Abbreviations

BMI: Body Mass Index
EMA: Ecological Momentary Assessment
F: Female
M: Male
MASLD: Metabolic-dysfunction associated steatotic liver disease
M/CA: Mexican and Central American
Y: Years
P: Participant
